# Does vitamin D play a significant role in type 2 diabetes?

**DOI:** 10.1186/s12902-015-0003-8

**Published:** 2015-02-26

**Authors:** Jayesh J Sheth, Ankna Shah, Frenny J Sheth, Sunil Trivedi, Mamta Lele, Navneet Shah, Premal Thakor, Rama Vaidya

**Affiliations:** Department of Biochemistry and Molecular Genetics, FRIGE’s Institute of Human Genetics, FRIGE House, Jodhpur Gam Road, Satellite, Ahmedabad, 380015 India; Unit of Endocrine and Metabolic Disorders, Kasturba Health Society, Medical Research Centre, Mumbai, 400056 India; Department of Diabetes and Endocrinology, Sterling Hospital, Ahmedabad, 380052 India; Gujarat Diabetic Association, Ahmedabad, 380007 India

**Keywords:** Diabetes, Vitamin D, HbA1c, HOMA-IR

## Abstract

**Background:**

Vitamin D deficiency reportedly is associated with type 2 diabetes (T2DM). We aim to examine whether 25-hydroxyvitamin D (25OHD) has clinically significant influence on hemoglobin glycation (HbA1c) and insulin resistance (HOMA-IR) in T2DM subjects.

**Methods:**

Present study was carried out in 912 subjects (429 T2DM cases and 483 non-diabetic controls) from Western India. The enrolled study subjects were investigated for biochemical parameters like FBS, PPBS, HbA1c, FI, HOMA-IR and 25OHD levels in blood.

**Results:**

Vitamin D deficiency was seen in 91.4% and 93.0% of T2DM cases and control subjects respectively. There was no association of serum 25OHD deficiency on HbA1c or HOMA-IR in T2DM cases (p = 0.057 & p = 0.257 respectively) and in control subjects (p = 0.675 & p = 0.647 respectively).

**Conclusion:**

Our findings suggests that though vitamin D deficiency is prevalent in T2DM and non-diabetic subjects, its role in hemoglobin glycation and insulin resistance could not be established.

## Background

Type 2 Diabetes Mellitus (T2DM) is the commonly seen endocrine disorder characterized by hyperglycemia [1]. The International Diabetes Federation (IDF) estimates around 61.3 million diabetic individuals (2011) in India that is further set to increase to 101.2 million with a global estimate of 552 million by the year 2030 [2]. There are several factors that seem to play a role in its development including genetic, lifestyle, environmental and nutritional conditions. Amongst nutritional factors, vitamin D is likely to have an important role either in glycemic control or in attenuating diabetic complications [3-5]. The probable mechanisms indicating the role of vitamin D in glucose homeostasis is likely to be through beta cell dysfunction and insulin resistance in cases with vitamin D deficiency [6,7]. A negative correlation between serum glucose and insulin levels with 25OHD and a positive correlation with insulin sensitivity has been observed in several human and animal model studies [6-9]. It has also been observed that vitamin D supplementation can improve insulin secretion and reduce insulin resistance in T2DM and non-diabetic subjects [7]. Thus, accumulating the evidence from several studies, vitamin D is likely to have a role in T2DM and Hb-glycation [10]. Nonetheless, the relationship between serum 25OHD with hemoglobin glycation (HbA1c) [11] and insulin resistance [12] in T2DM has not been extensively studied except for one report from US adult population with T2DM and non-diabetic subjects [11] and none from Western Indian population. Hence, present study was proposed to examine the association of vitamin D (25OHD) levels with HbA1c and HOMA-IR in this population.

## Methods

### Subjects

The present prospective cross sectional study was conducted after taking Foundation for Research in Genetics and Endocrinology (FRIGE) institutional ethics committee approval for 912 subjects (429 T2DM cases and 483 non-diabetic controls) of both genders with age ranging from 25 to 86 years. They were selected between April, 2012 to July, 2014 from the Out Patient Departments (OPD) of various diabetologists and through weekly camps at the institute. The participants of the current study were enrolled from an urban metropolitan western Indian population. The inclusion criteria for T2DM subjects were (a) age (≥25 years); (b) duration of diabetes (≥6 months); and (c) plasma glucose levels (FBS ≥ 126.0 mg/dl) [13]. Control subjects recruited in the study were non-diabetic with an inclusion criterion of (a) age (≥25 years); (b) HbA1c level ≤ 6.5%; and (c) plasma glucose levels (FBS ≤ 110.0 mg/dl). An institutional ethical committee approval and written informed consent was obtained from all the subjects. Subjects with Type 1 Diabetes, lactating and pregnant mothers, those with concomitant illness and intake of vitamin D supplements were excluded from the study. Anthropometric parameters such as age, BMI, waist circumference, waist-hip ratio and duration of diabetes (in T2DM subjects) were recorded for all the study subjects.

Serum levels of 25OHD were stratified into normal (≥30 ng/ml), insufficient (≥20 to <30 ng/ml) and deficient (<20 ng/ml) [14]. Subsequently, for this study, we considered all 25OHD values of <20 ng/ml as vitamin D deficient and ≥20 ng/ml as non-deficient, with regard to the biological interval used in our laboratory.

### Anthropometric indices

Weight was measured with light clothes without shoes using digital scale (to the nearest 0.1 kg). Height was measured without shoes using stadiometer (to the nearest 0.1 cm). Waist circumference was measured (to the nearest 0.1 cm). BMI was calculated using the equation “BMI = weight/height^2^ (kg/meter^2^)”.

### Sample collection and handling

Blood samples were collected in fluoride and serum vaccutainers between 8 to 11 AM after 12 hours fasting for biochemical assays [fasting blood sugar (FBS), glycosylated hemoglobin (HbA1c), fasting insulin (FI), HOMA-IR, and vitamin D (25OHD) estimation]. Blood was collected again post 2 hours of consuming non-standardized meal for post prandial blood sugar (PPBS) estimation for T2DM subjects while non-diabetic control subject were not investigated for PPBS. Serum was separated within 30-45 minutes, aliquoted and stored at -20°C till analysis.

### Biochemical investigations

All subjects were investigated for biochemical parameters such as FBS & PPBS, HbA1c, FI, HOMA-IR and 25OHD. FBS and PPBS were determined from plasma by commercially available biochemical kits using an auto-analyzer system (BTS 330, Biosystem, Spain). HbA1c level were measured using affinity assay method by NycoCard reader-II (Axis-Shield, Norway) from whole blood. FI levels were measured by Immune Radiometric Assay (IRMA) using commercial kit (Immunotech, France) in γ-counter system (PC-RIA.MAS, Stratec Biometrical System AG, Germany). Insulin resistance was calculated by the Homeostasis Model of Assessment-Insulin Resistance Index (HOMA-IR) [as (FI × FBS)/405, where FI: Fasting Insulin and FBS: Fasting Blood Sugar] [15]. Serum concentrations of 25-hydroxyvitamin D (25OH-D2 and 25OH-D3) were assessed by ELISA method performed on micro-titer plates using ELISA kit (Demeditec Diagnostics GmbH, Germany).

### Statistical analysis

The subjects were recruited based on consecutive sampling technique. The sample size yielded a margin of error of 3.1% of the study with confidence limit of 95% with on-line calculator [16]. False positive error rate/Type I error was 0.05. Comparisons of all variables like FBS, PPBS, HbA1c, fasting insulin and HOMA-IR were performed with Student’s *t*-test. Value of HbA1c was given as percentage of total hemoglobin and values of all other glucose parameters were given in mg/dl. All values are expressed as mean ± standard deviation. Linear regression analysis was also performed. The analysis was carried out with IBM-SPSS v15.0. p values (two tailed, <0.05) were considered as statistically significant.

## Results

Vitamin D deficiency (<20 ng/ml) was a common finding in this cohort affecting approximately 91.4% and 93.0% of T2DM cases (Group-1) and control subjects (Group-2) respectively. Hence, both the groups were further subdivided in to vitamin D deficient (25OHD <20 ng/ml) and non-deficient (25OHD ≥20 ng/ml). Anthropometric indices such as age, BMI, waist circumference, waist-hip ratio, duration of diabetes, and biochemical parameters such as FBS, HbA1c, FI and HOMA-IR were compared between the vitamin D-deficient and non-deficient group in addition to case and control subjects where notably no significant difference was observed as shown in Table 1. All the patients and controls were city dwellers; majority of them were office workers or housewives and connected to non-strenuous life style with minimal aerobic exercise during the day. Increased BMI (>25 kg/m^2^) was seen in 64.5% (277/429) of T2DM subjects. Higher BMI was observed in 65.2% (255/391) of vitamin D deficient (low serum 25OHD) patients as opposed to 60.0% (21/35) of non-deficient (normal serum 25OHD) T2DM subjects. Similarly, increased waist circumference (Females >80 cms, Males: >90 cms) was above upper limit of normal in 81.5% (350/429) of subjects; increased in 81.9% (319/390) of vitamin D deficient versus 85.7% (30/35) of non-deficient T2DM subjects. The waist : hip ratio (WHR) also followed a similar pattern of increased WHR (Females: >0.85, Males: >0.90) in 83.2% (357/29) of T2DM subjects; 83.8% (328/391) of vitamin D deficient vs 80.0% (28/35) of vitamin D non-deficient T2DM subjects.

**Table 1:** **Baseline demographic and biochemical indices of T2DM cases and control subjects according to their vitamin D status**

**Parameters (T = 912)**	**T2DM Cases (N = 429)**			**Non-diabetic Controls (N = 483)**		
	**Serum 25OHD** **<20 ng/ml**	**Serum 25OHD** **≥20 ng/ml**	**p value**	**Serum 25OHD** **<20 ng/ml**	**Serum 25OHD** **≥20 ng/ml**	**p value**
	**Mean ± SD**	**Mean ± SD**		**Mean ± SD**	**Mean ± SD**	
	**(n = 392)**	**(n = 37)**		**(n = 449)**	**(n = 34)**	
**Anthropometric indices**						
**Age (Years)**	56.37 ± 10.34	57.79 ± 12.19	0.44	48.36 ± 12.78	51.44 ± 13.51	0.18
**BMI (Kg/m** ^**2**^ **)**	27.17 ± 5.14	26.50 ± 3.93	0.44	25.78 ± 4.54	26.68 ± 5.20	0.27
**WC (cm)**	96.99 ± 10.68	94.31 ± 9.21	0.14	92.10 ± 10.71	91.59 ± 10.61	0.78
**WHR**	0.94 ± 0.10	0. 93 ± 0.07	0.55	0.91 ± 0.07	0.89 ± 0.08	0.11
**Duration of T2DM (Years)**	8.30 ± 7.26	11.23 ± 10.55	0.03*	-	-	-
**Biochemical indices**						
**FBS (mg/dl)**	150.43 ± 64.51	138.80 ± 47.09	0.29	88.18 ± 12.11	87.88 ± 13.51	0.89
**PPBS (mg/dl)**	190.72 ± 70.50	187.42 ± 76.74	0.79	-	-	-
**HbA1c (%)**	8.36 ± 1.79	8. 26 ± 2.21	0.75	5.67 ± 0.49	5.67 ± 0.54	0.82
**FI (uIU/ml)**	11.29 ± 6.95	8.27 ± 4.17	0.01*	12.11 ± 5.45	10.00 ± 5.00	0.03*
**HOMA-IR**	3.93 ± 3.62	3.04 ± 2.24	0.04*	2.71 ± 1.60	2.20 ± 1.24	0.08

By student’s *t*-test, *: Significant, SD: Standard deviation.

BMI: Body mass index, WC: Waist circumference, WHR: Waist-Hip-Ratio.

FBS: Fasting Blood Sugar, PPBS: Post-Prandial Blood Sugar, HbA1c: Glycated hemoglobin, FI: Fasting Insulin, HOMA-IR: Homeostasis Model of Assessment-Insulin Resistance Index.

Vitamin D-Deficient: 25OHD <20ng/ml.

Vitamin D-Non-deficient: 25OHD ≥20ng/ml.

**Figure 1:**
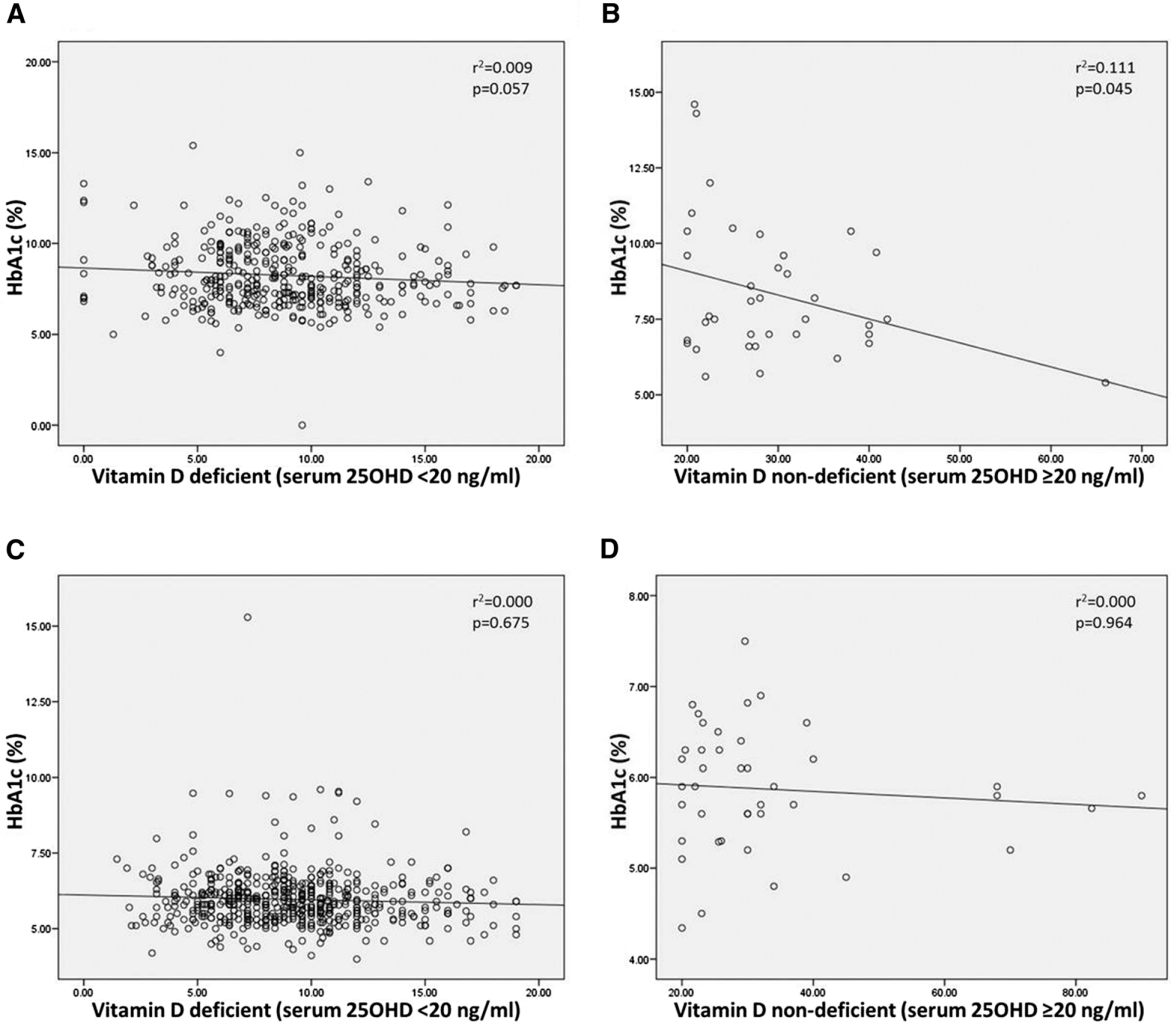
**Relationship between vitamin D and HbA1c in deficient and non-deficient T2DM and Controls. A.** Relationship between vitamin D deficient T2DM cases and HbA1c. **B.** Relationship between vitamin D non-deficient T2DM cases and HbA1c. **C.** Relationship between vitamin D deficient Controls and HbA1c. **D.** Relationship between vitamin D non-deficient Controls and HbA1c.

Results from linear regression analysis indicate that vitamin D deficiency did not have any statistically significant association with HbA1c levels in both the groups [T2DM: r^2^ = 0.009, p = 0.057 (95% CI = -0.095 to 0.003) and Control: r^2^ = 0.000, p = 0.675 (95% CI = -0.016 to 0.010)] respectively. Similarly, no association was detected in subjects of both the groups with normal vitamin D levels and HbA1c levels [T2DM: r^2^ = 0.111, p = 0.045 (95% CI = -0.156 to -0.002) and Control: r^2^ = 0.000, p = 0.964 (95% CI = -0.011 to 0.010)] respectively as shown in Figure 1.

**Figure 2:**
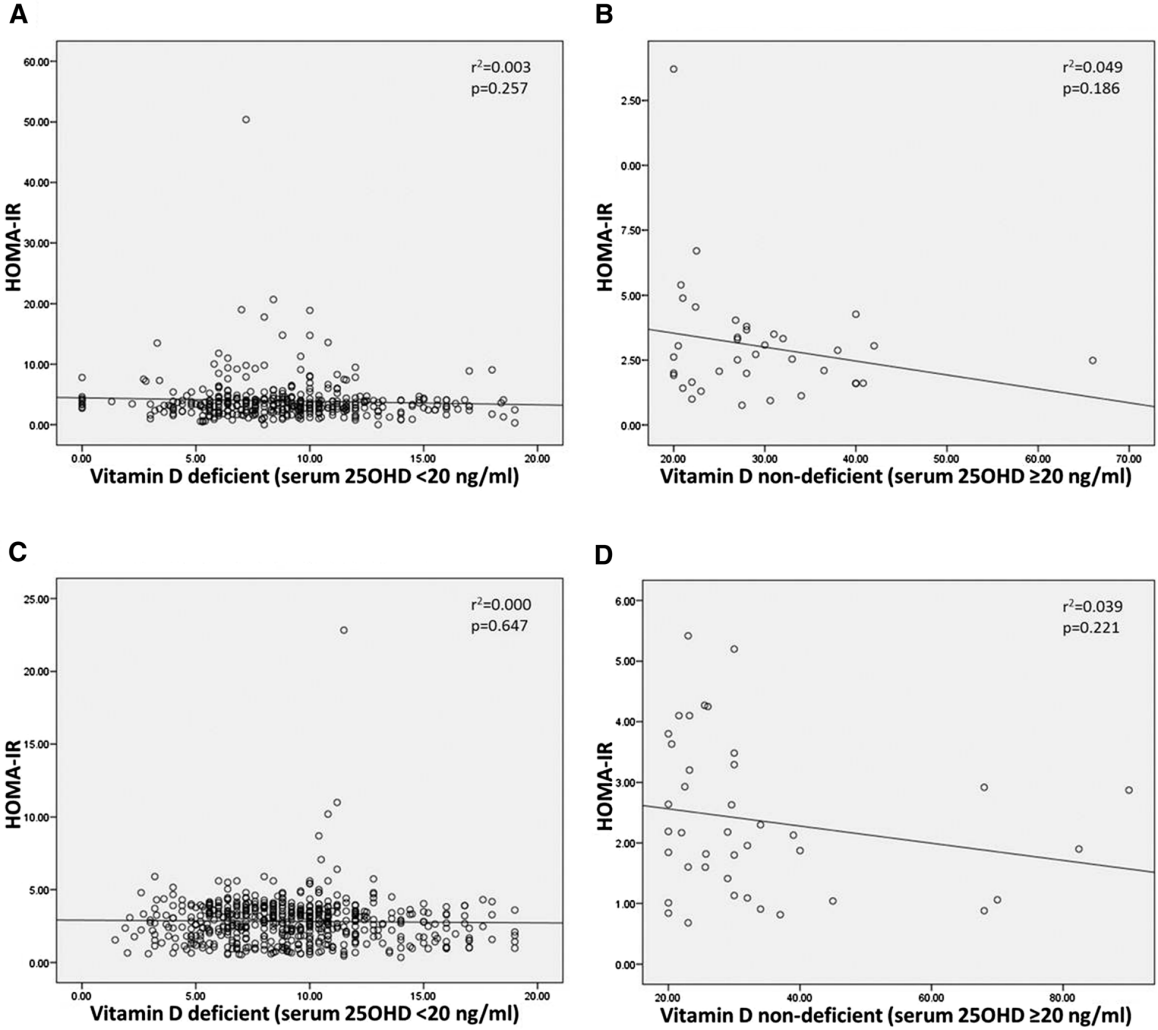
**Relationship between vitamin D and HOMA-IR in deficient and non-deficient T2DM and Controls. A.** Relationship between Vitamin D deficient T2DM cases and HOMA-IR. **B.** Relationship between Vitamin D non-deficient T2DM cases and HOMA-IR. **C.** Relationship between Vitamin D deficient Controls and HOMA-IR. **D.** Relationship between Vitamin D non-deficient Controls and HOMA-IR.

Additionally, linear regression analysis results between vitamin D levels and HOMA-IR indicate that vitamin D deficiency did not have any statistically significant association with HOMA-IR in both the groups [T2DM: r^2^ = 0.003, p = 0.257 (95% CI = -0.157 to 0.042) and Control: r^2^ = 0.000, p = 0.647 (95% CI = -0.053 to 0.035)] respectively. Likewise, no association was seen in subjects of both the groups with normal vitamin D levels and HOMA-IR levels [T2DM: r^2^ = 0.049, p = 0.186 (95% CI = -0.135 to 0.027) and Control: r^2^ = 0.039, p = 0.221 (95% CI = -0.034 to 0.013)] respectively as described in Figure 2.

## Discussion

The increasing incidence of T2DM is taking a great toll of health resources. This has laid a number of research studies related to life style, environmental and nutritional factors in an attempt to ameliorate its burden. The diverse effect of vitamin D on glucose and calcium homeostasis [17] has made it an ideal contender to know its role in glycemic control in T2DM. India being a vast tropical country geographically spreading from 8.4° N latitude to 37.6° N latitude, it is expected that sufficient sunlight is received throughout the year [14,18,19]. Regardless of this vitamin D deficiency has been observed more commonly in earlier studies from India [14,19]. The present study has also shown a higher incidence (91.4%) of vitamin D deficiency in overall recruited subjects indicating that both T2DM (91.4%) subjects and non-diabetic control subjects (93.0%) were equally deficient. This is in accordance with other studies demonstrating low serum vitamin D levels in 70% to 100% populations across India [14,18,19]. This is likely to be due to increased skin pigmentation, low exposure to direct sunlight, obesity and malabsorption, as has been observed by several studies from India [14,18,19]. It has been argued by Lo *et al.* that to meet an adequate requirement of vitamin D, people in India require sun exposure almost double than Caucasians due to increased skin pigmentation [20-22]. Life style factors like in-door working or working in close environment with minimum sun exposure is also likely for high prevalence of vitamin D deficiency in our population. Normal office hours in India are usually from 11 am to 7 pm while maximum sun exposure and absorption is between 11 am to 2 pm with an UV index of 7-9 required for conversion of 7-dehydrocholesterol to pre-vitamin D_3_ [18]. But this seems to be unrealistic as being a tropical country summers in India are very hot, forcing most of its people to stay indoor during this time. This results in low exposure to the sunlight contributing for very low vitamin D status in our population.

Study by Macdonald *et al.* [23] has suggested that vitamin D status might not be the only marker of ill health, but also an indicator of lifestyle of an individual like indoor working with restrictions of sunlight exposure, low mobility, dietary habits that might affect long-term health.

Though most of the observational studies cannot demonstrate the cause and effect related to vitamin D, lower vitamin D status might be a reflection of sedentary lifestyle and chronic non-specific illness. It can also be argued that the people with normal levels of vitamin D are in overall good health with better lifestyle and normal weight [24]. Although in a review by Pittas *et al.* an association between T2DM and low vitamin-D levels has been demonstrated [10]. Nonetheless, vitamin-D supplementation was not found to be effective in reducing HbA1c as stated by Melville in his news report [25]. Our finding of absence of significant association of hemoglobin glycation with vitamin D further questions its definitive role in T2DM except for poor lifestyle in our overall population. Luo *et al*. also showed that within T2DM subjects, regardless of a common finding of vitamin D deficiency, low vitamin D is associated neither with increased prevalence of the metabolic syndrome, nor is there any association with glycemic control [17].

Several mechanisms like activation of vitamin D receptor and calcium homeostasis involving impaired pancreatic-β cell function and insulin resistance in T2DM have been suggested [10]. This has been confirmed by *in vitro* studies in animal models suggesting its role in improving insulin sensitivity and secretion [10,17,26], though the associations between 25OHD, glucose homeostasis, and insulin resistance in humans seems to be inconsistent [26]. Also a number of studies have shown a consistent inverse association between vitamin D level or vitamin D intake on the incidence of T2DM [3,4], but our study could not demonstrate such relationship. Similar observation has been made in studies from New Zealand overweight adult population and British Caucasians demonstrating a weak relationship between HbA1c and vitamin D levels [27,28]. In a recent study by Davidson *et al.* on subjects unknown to have diabetes failed to demonstrate the effect of vitamin D supplementation to predict the development of diabetes in pre-diabetic and those with low vitamin D level compared to placebo group [29]. As with the progression in the duration of T2DM, the β-cell reserve attenuates [30], but in our study we could not observe significant association between HbA1c and HOMA-IR with 25OHD taking into account with the duration of T2DM.

An Australian study by Elkassaby *et al.* [31] recently observed a transient improvement in glycemia in T2DM with oral D_3_ supplementation without change in either HbA1c or beta cell function and concluded that high dose D_3_ has a little or no therapeutic benefit. A similar study from UAE [32] has also reported no significant change in HbA1c levels after six month of supplementation with vitamin D_3_ in vitamin D-deficient obese T2DM patients of Emirati population. A study performed on Indian subjects residing in New Zealand [33] has shown a significant correlation between insulin sensitivity and IR and decrease in FI with vitamin D supplementation while there was a significant negative correlation between HbA1c and vitamin D levels due to supplementation in South Asian subjects in UK [34]. Though, both the studies were carried out on relatively smaller populations as compared to the one under report. Furthermore, in a review by Pittas *et al.* it was shown at least in seven trials that vitamin D supplementation has no role on glycemic measures and HOMA-IR (as an indicator of insulin resistance) in participants with normal glucose tolerance [35]. In addition, no effect of vitamin D supplementation was evident in four out of five trials (with participants from normal glucose tolerance) that reported insulin resistance as an outcome [35]. A review study by George *et al.* again argued against vitamin D supplementation for improving glycemic control and insulin resistance in T2DM and non-diabetic subjects [36]. Moreover, a recent study by Al-Shoumer *et al.* demonstrated the prevalence of vitamin D deficiency in insulin resistant T2DM and normal subjects, where insulin resistance was not found to be influencing the status of vitamin D [37]. Further confirming this, Kampmann *et al.* and Witham *et al.* showed that improvement in vitamin D status may increase insulin secretion but did not improve insulin resistance and HbA1c in patients with T2DM [38,39]. This is in concordance with our findings that vitamin D levels did not show any significant linear association with HOMA-IR status in T2DM cases as well as control subjects. This is possibly because the inflammatory mechanisms are extremely stimulated by the diabetic milieu or the β-cell dysfunction, and insulin resistance is more severe and less reversible by extended duration of diabetes as explained by Luo *et al.* [17].

### Limitation

The study was a cross sectional study, carried out on urban metropolitan subjects where rural subjects were not included. Moreover, the method used to measure vitamin D levels was ELISA method which is less sensitive to LCMS/MS method.

## Conclusion

Though vitamin D deficiency is prevalent in T2DM and non- diabetic control subjects, its relationship in glycation control or insulin resistance in T2DM subjects could not be confirmed in our population. This is potentially an important finding for public health, demonstrating that improvement in vitamin D status is not the only factor responsible for better health of the individuals but lifestyle and dietary changes seem to play a role which will improve the overall health including hemoglobin glycation and insulin resistance along with vitamin D levels. Whether vitamin D supplementation can delay the onset of diabetes remains to be recognized. Therefore, future studies to clarify the efficacy of vitamin D supplementation in preventing diabetes and pre-diabetes are warranted, especially in populations at high risk.

## Abbreviations

T2DM: Type 2 Diabetes Mellitus
HbA1c: Glycated Hemoglobin
HOMA-IR: Homeostasis Model of Assessment-Insulin Resistance Index
FBS: Fasting Blood Sugar
PPBS: Post prandial Blood Sugar
IDF: International Diabetes Federation
BMI: Body Mass Index
FI: Fasting Insulin
